# DAAM2 is elevated in the circulation and placenta in pregnancies complicated by fetal growth restriction and is regulated by hypoxia

**DOI:** 10.1038/s41598-021-84785-7

**Published:** 2021-03-10

**Authors:** Natasha de Alwis, Sally Beard, Natalie K. Binder, Natasha Pritchard, Tu’uhevaha J. Kaitu’u-Lino, Susan P. Walker, Owen Stock, Katie Groom, Scott Petersen, Amanda Henry, Joanne M. Said, Sean Seeho, Stefan C. Kane, Lisa Hui, Stephen Tong, Natalie J. Hannan

**Affiliations:** 1 Therapeutics Discovery and Vascular Function in Pregnancy Group, Mercy Hospital for Women, Heidelberg, VIC 3084 Australia; 2 Translational Obstetrics Group, Mercy Hospital for Women, Heidelberg, VIC 3084 Australia; 3 Mercy Perinatal, Mercy Hospital for Women, Heidelberg, VIC 3084 Australia; 4 Northern Health, Epping, VIC 3076 Australia; 5 Department of Obstetrics and Gynaecology, University of Melbourne, Melbourne, VIC Australia; 6 Liggins Institute, University of Auckland, Auckland, 1023 New Zealand; 7 Centre for Maternal Fetal Medicine, Mater Mothers’ Hospital, South Brisbane, QLD 4101 Australia; 8 School of Women’s and Children’s Health, UNSW Medicine, University of New South Wales, Sydney, Australia; 9 Maternal Fetal Medicine, Joan Kirner Women’s & Children’s Sunshine Hospital, St Albans, VIC 3021 Australia; 10 The University of Sydney Northern Clinical School, Women and Babies Research, St Leonards, NSW 2065 Australia; 11 Department of Maternal Fetal Medicine, Royal Women’s Hospital, Parkville, VIC 3052 Australia

**Keywords:** Intrauterine growth, Predictive markers, Genetics research, Gene expression

## Abstract

Previously, we identified increased maternal circulating *DAAM2* mRNA in pregnancies complicated by preterm fetal growth restriction (FGR). Here, we assessed whether circulating *DAAM2* mRNA could detect FGR, and whether the *DAAM2* gene, known to play roles in the Wnt signalling pathway is expressed in human placenta and associated with dysfunction and FGR. We performed linear regression analysis to calculate area under the ROC curve (AUC) for *DAAM2* mRNA expression in the maternal circulation of pregnancies complicated by preterm FGR. *DAAM2* mRNA expression was assessed across gestation by qPCR. DAAM2 protein and mRNA expression was assessed in preterm FGR placenta using western blot and qPCR. *DAAM2* expression was assessed in term cytotrophoblasts and placental explant tissue cultured under hypoxic and normoxic conditions by qPCR. Small interfering RNAs were used to silence *DAAM2* in term primary cytotrophoblasts. Expression of growth, apoptosis and oxidative stress genes were assessed by qPCR. Circulating *DAAM2* mRNA was elevated in pregnancies complicated by preterm FGR [p < 0.0001, AUC = 0.83 (0.78–0.89)]. Placental *DAAM2* mRNA was detectable across gestation, with highest expression at term. DAAM2 protein was increased in preterm FGR placentas but demonstrated no change in mRNA expression. *DAAM2* mRNA expression was increased in cytotrophoblasts and placental explants under hypoxia. Silencing *DAAM2* under hypoxia decreased expression of pro-survival gene, *BCL2* and oxidative stress marker, *NOX4*, whilst increasing expression of antioxidant enzyme, *HMOX-1*. The increased DAAM2 associated with FGR and hypoxia implicates a potential role in placental dysfunction. Decreasing *DAAM2* may have cytoprotective effects, but further research is required to elucidate its role in healthy and dysfunctional placentas.

## Introduction

The placenta is a unique organ developed in pregnancy, acting as the interface between the maternal and fetal systems. Healthy placental development creates a high flow, low resistance blood delivery system to facilitate the supply of oxygen and nutrients and removal of waste products. This exchange is essential for optimal fetal growth, and as such, appropriate placental development is crucial to establish a healthy pregnancy. Aberrant placentation can progress to serious pregnancy complications such as fetal growth restriction, which is responsible for significant perinatal morbidity and mortality^1^.

In the first trimester of pregnancy, cytotrophoblasts, a cell type unique to the placenta regulate the remodelling of maternal spiral arteries. The cytotrophoblasts replace endothelial cells, whilst simultaneously expanding the vessel lumen, as well as forming trophoblast plugs^2,3^. These plugs create a low oxygen environment for initial placental and fetal development. When the trophoblast plugs disintegrate towards the end of the first trimester, the remodelled arteries can provide a high flow, low resistance blood supply to the placenta and fetus, restoring oxygen levels^4^.

Impaired trophoblast invasion and remodelling of the maternal spiral arteries causes placental insufficiency, resulting in impaired circulation to the placenta. Blood flow can become pulsatile, damaging the fragile placental tissue resulting in placental ischaemia and increased oxidative stress^5^. Furthermore, oxygen delivery may be compromised, generating variations in oxygen tension and periods of low oxygen referred to as placental hypoxia^6^. This failure in placental function can severely affect fetal development^7^.

Fetal growth restriction is a serious pregnancy complication where a fetus fails to reach its growth potential due to inadequate nutrient supply. The most common cause is uteroplacental insufficiency, where dysfunctional placentation and chronic hypoxia impair fetal growth and development^5^. Fetal growth restriction is associated with an increased risk of major perinatal injury, cardiovascular, respiratory and neurological morbidities and major risk of mortality, with early onset cases being most serious^8–10^. Despite being one of the most severe complications of pregnancy, with up to 45% of non-anomalous stillbirths associated with fetal growth restriction^11^, there are still no treatments that improve placental insufficiency nor accurate means of early detection of placental dysfunction preceeding severe impairment to fetal growth. There is an urgent clinical need to develop better tests for placental insufficiency to decrease perinatal morbidity and mortality.

In a recent multi-center cohort study, we used next generation sequencing to measure cell-free RNA in the blood of pregnant women with preterm fetal growth restriction, fetal acidemia in utero, and stillbirth. Our study identified significantly altered mRNA signatures in the maternal circulation where there was placental insufficiency, preterm fetal growth restriction and fetal hypoxia^12^. Dishevelled Associated Activator of Morphogenesis 2 (*DAAM2*) was one of the most differentially regulated genes identified. *DAAM2* mRNA was elevated in the circulation of women with preterm fetal growth restriction. Whether these circulating mRNAs originated from the dysfunctional placenta was not established in that study.

DAAM2 is known for its role in the Wnt signalling pathway^13,14^. Recently, the first study to report on *Daam2* in the placenta demonstrated a role in placental vascularization and establishment of the maternal–fetal blood supply in mice^15^. However, there are no published studies investigating *DAAM2* expression or function in the human placenta.

In this study, we aimed to assess whether *DAAM2* was expressed in the human placenta and whether gene expression or protein production was altered by gestation, or placental dysfunction associated with preterm fetal growth restriction. We also set out to explore possible functional roles for *DAAM2* in the placenta related to growth and dysfunction.

## Results

### DAAM2 is increased in the circulation of pregnancies complicated by fetal growth restriction

Using next-generation sequencing, we initially discovered *DAAM2* expression was increased in the maternal circulation of pregnancies complicated by preterm fetal growth restriction (FGR) in the FOX study cohort^12^. The majority of the fetal growth restricted cases were significantly growth restricted with a median birthweight centile (using intrauterine fetal charts^16^) of 0.1 (interquartile range 0.0–0.4; see Table 1 for baseline clinical characteristics of women in the FOX study).

**Table 1:** **Patient characteristics for cases of Fetal Growth Restriction (FGR) and control cohorts as part of the FOX study.**

Characteristics	FGR cases (n = 128)	Controls (n = 42)	*P*
Maternal age, years	32 (6)	30 (6)	0.13
Nulliparity	80 (63%)	38 (45%)	0.013
Body-mass index, kg/m^2^	27 (6)	24 (5)	0.0009
Smoking during pregnancy	17 (13%)	6 (7%)	0.16
Diabetes during pregnancy	15 (12%)	8 (10%)	0.60
Chronic hypertension	12 (9%)	0 (0%)	0.004
Preeclampsia	63 (49%)	3 (4%)	< 0.00001
Absent or reversed end diastolic flow in umbilical artery	58 (45%)	–	–
Median gestational age at blood sampling (wks)	30.5 (28.6–32.1)	30.0 (28–32.1)	0.21
Gestational age at delivery (wks)	30.5 (28.6–32.1)	39.4 (39–40.2)	< 0.00001
Birthweight, g	1023 (315)	3594 (480)	< 0.00001
Birthweight centiles, corrected for gestation^a^ (median and interquartile range)	0.1 (0.0–0.4)	40.8 (27.9–60.9)	< 0.00001
Male sex	71 (55%)	46 (55%)	0.92
Umbilical artery pH, median	7.27 (7.22–7.3)	–	
Umbilical artery pH < 7.2	22 (17.2%)	–	
Neonatal deaths within 42 days of birth	2 (2%)	0 (0%)	0.42

Data are n (%), mean (SD), or median (IQR). Comparison between FGR cases and gestation matched controls is by chi squared analysis and *t*-test. Each control contributed two blood samples for the analysis, at 28 and 32 weeks gestation. This was done to correct for possible changes in RNA concentrations across gestational age.

^a^We used fetal weight reference charts to determine centiles (Hadlock formula, except fetal sex was corrected for).

**Figure 1:**
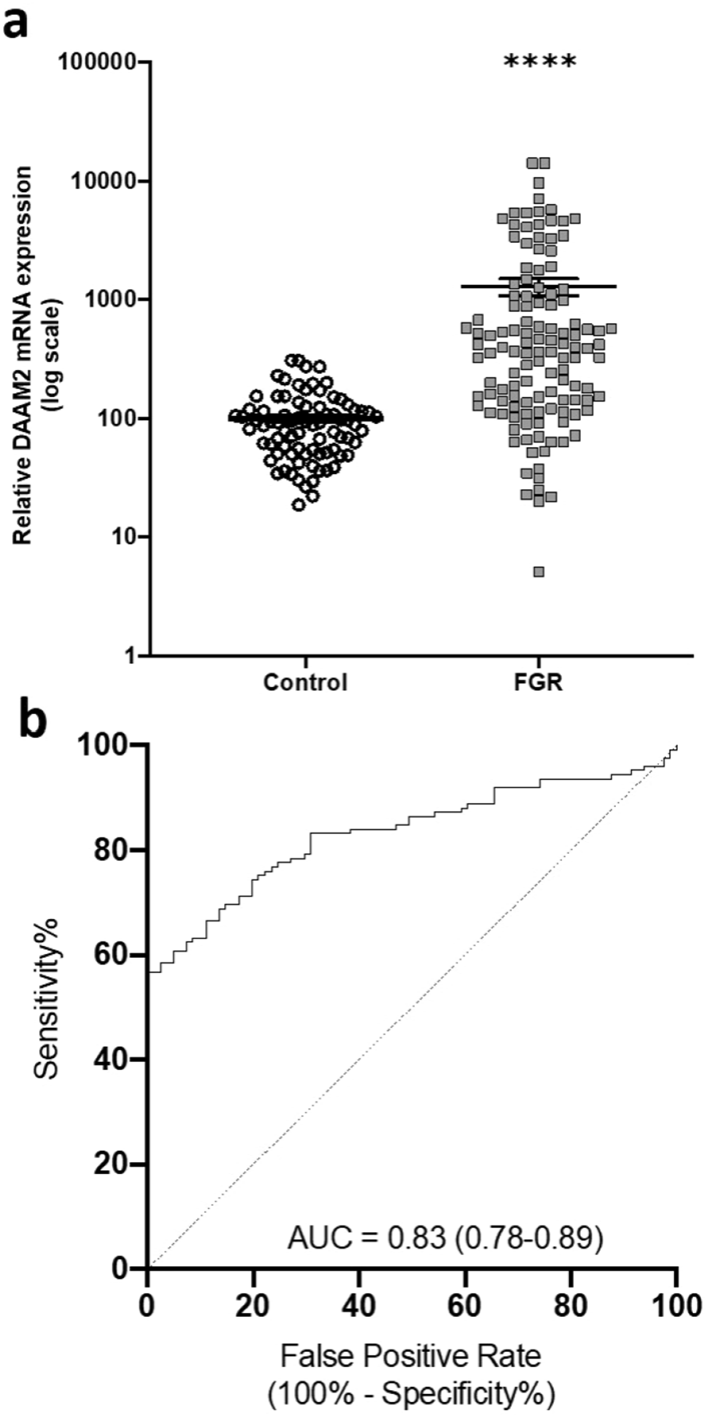
Expression of *DAAM2* mRNA in the maternal circulation from pregnancies complicated by preterm fetal growth restriction (FOX Study). *DAAM2* mRNA data is expressed as log expression; error bars are median ± IQR. AUC = 0.83; 95% Confidence Interval (0.78–0.89). ****p < 0.0001.

Here, in the current study we have further analysed the quantitative PCR data presented in our previous study^12^ to specifically determine the ability of *DAAM2* to detect preterm FGR. We log-transformed circulating *DAAM2* mRNA expression, demonstrating a highly significant increase (p < 0.0001) in circulating *DAAM2* mRNA in pregnancies complicated by preterm fetal growth restriction (Fig. 1a) compared to gestation-matched controls. Additionally, we examined whether maternal circulating *DAAM2* mRNA was altered between cases of preterm FGR where fetal acidemia (associated with increased risk of perinatal death^17^) was apparent (determined by an umbilical artery blood pH < 7.2 (indicating acidosis) versus pH ≥ 7.2 (not acidotic)). We did not detect further altered expression of *DAAM2* in the circulation with fetal hypoxia (Supplementary Fig. S1).

To determine whether *DAAM2* RNA could provide a useful test, we performed logistic regression analysis. This provided a test with an area under the receiver operating characteristic (ROC) curve of 0.83 (Fig. 1b). At a specificity of 90.1% (i.e. a 10% screen positive rate) the test had 63.2% sensitivity in identifying preterm fetal growth restriction, with a positive likelihood ratio of 6.4. Thus, circulating *DAAM2* mRNA expression demonstrated potential to highlight pregnancies at serious risk of preterm fetal growth restriction and may be useful in a multi-marker test. However, use as a lone marker would require further validation, with comparison to current clinical detection of high-risk pregnancies.

We also found no change in *DAAM2* expression when we sub-analysed the cases by coexistent gestational hypertension or preeclampsia (Supplementary Fig. S2). Assessment between 28 and 32 weeks in the control samples revealed no significant increase in DAAM2 expression in the maternal circulation with advancing gestation (data not shown). *DAAM2* mRNA was not altered by fetal gender (data not shown).

### DAAM2 is expressed in human placenta and increases with advancing gestation

**Figure 2:**
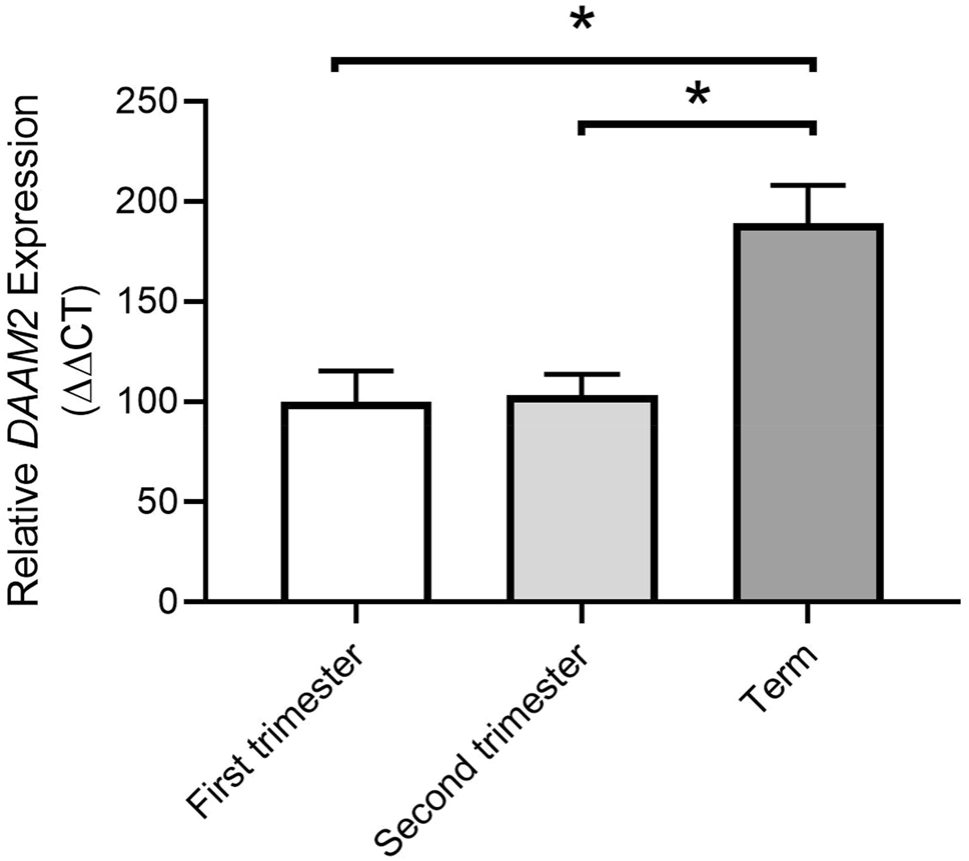
*DAAM2* expression in placentas from first trimester, second trimester and term gestation. Expression of *DAAM2* is significantly increased at term compared to first trimester and second trimester. There is no change in *DAAM2* expression between first trimester and second trimester. Data presented as fold change from first trimester, mean ± SEM. *p < 0.05. First trimester; n = 6, 7–9 weeks. Second trimester; n = 4, 24–29 weeks. Term; n = 9; 38–39 weeks.

*DAAM2* expression was identified in human placental tissue at all gestations examined (first trimester, second trimester and term). There was no significant change in expression between first trimester (7–10 weeks) and second trimester (24–29 weeks) samples. However, a significant increase in expression was observed between the term and first trimester samples (p = 0.008; Fig. 2). *DAAM2* expression was also significantly higher at term compared to second trimester gestation (p = 0.034; Fig. 2). Thus, placental *DAAM2 e*xpression increases with advancing gestation.

### DAAM2 protein is increased in placental tissue from pregnancies affected by fetal growth restriction

**Figure 3:**
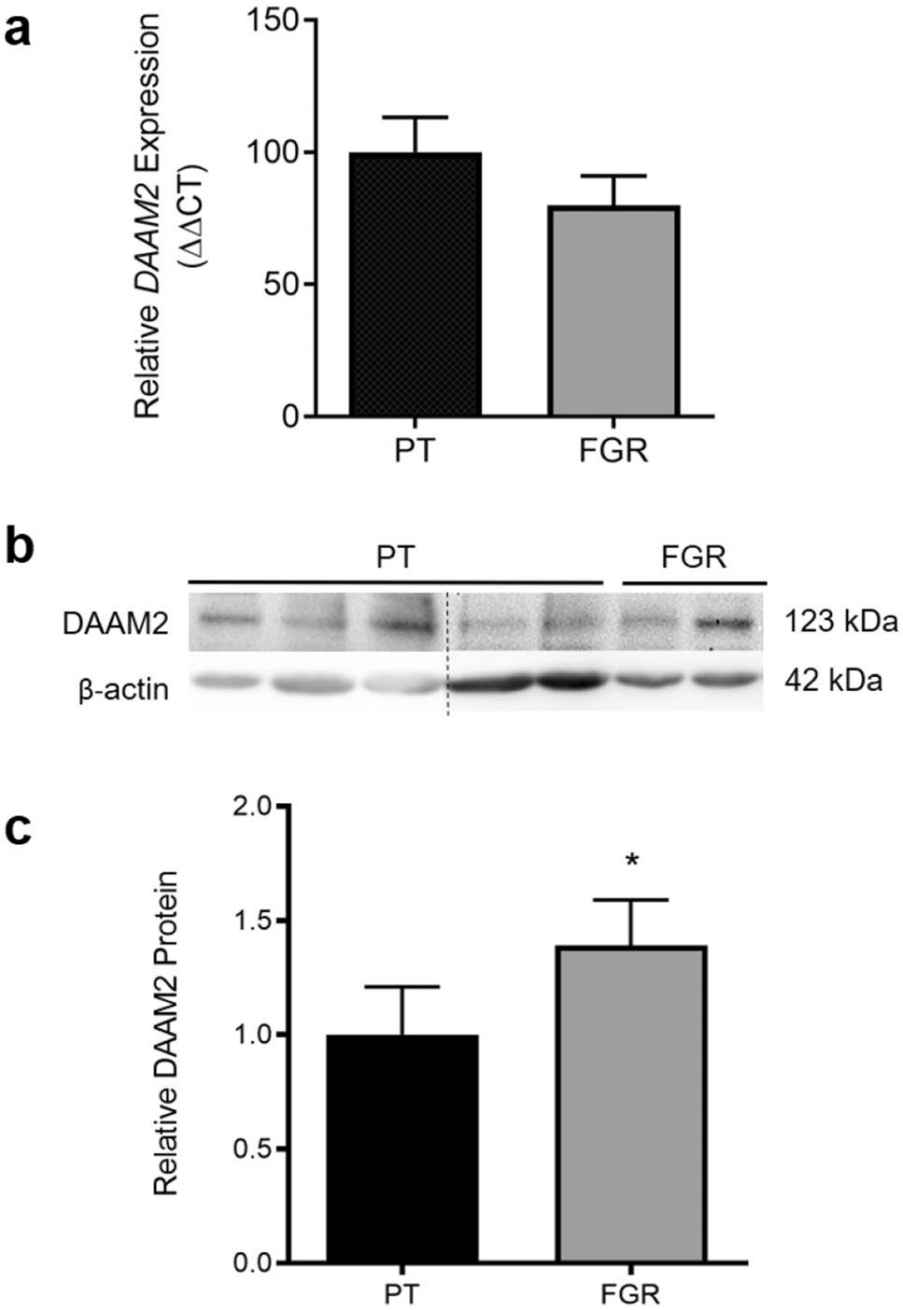
*DAAM2* mRNA and protein in preterm placental tissue (≤ 34 weeks). (**a**) *DAAM2* mRNA expression assessed by qPCR. (**b**) Representative western blot and (**c**) densitometric analysis of DAAM2 protein. *DAAM2* mRNA expression was not significantly changed in fetal growth restricted (FGR; n = 14) tissue compared to preterm control (PT; n = 10) tissue. Relative levels of DAAM2 protein were significantly higher in fetal growth restricted (n = 16) placental tissue compared to preterm control (n = 9) tissue. Protein and mRNA data presented as fold change from control ± SEM. *p < 0.05. Full western blot images are presented in Supplementary Fig. S3.

There was no significant difference in *DAAM2* mRNA expression in placental tissue from pregnancies complicated by preterm fetal growth restriction (≤ 34 weeks gestation) compared to gestation-matched preterm control placenta (Fig. 3a). However, placental DAAM2 protein was significantly increased in pregnancies complicated by preterm fetal growth restriction, compared to gestation-matched control placentas (p = 0.049; Fig. 3b,c, Supplementary Fig. S3).

### DAAM2 expression is increased under hypoxia in term placental tissue and isolated cytotrophoblasts

**Figure 4:**
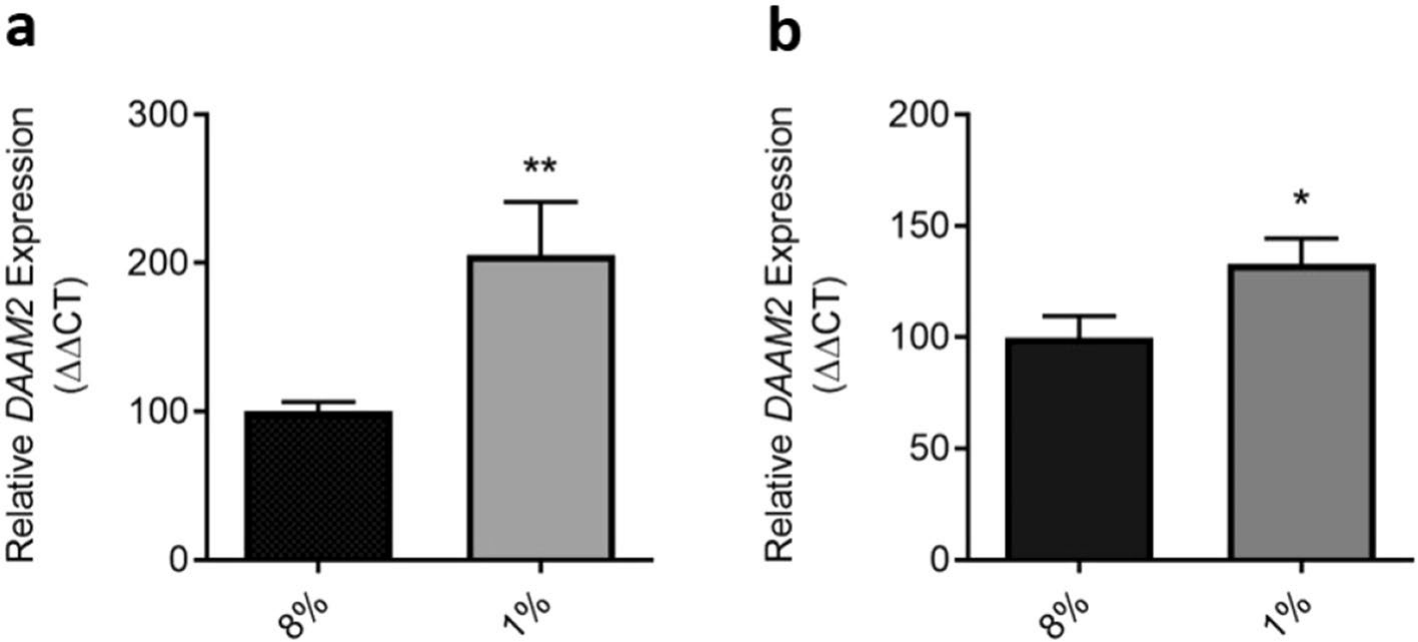
Expression of *DAAM2* mRNA in primary cytotrophoblasts and placental explant tissue under normoxic (8% O_2_) and hypoxic (1% O_2_) conditions. Cytotrophoblast (**a**) and placental explant (**b**) expression of *DAAM2* mRNA is significantly upregulated under hypoxic conditions, compared to normoxic control. Data presented as fold change from control ± SEM. *p < 0.05, **p < 0.01. n = 4–5 experimental replicates (each sample from a different patient), with each experiment performed in triplicate.

Under hypoxic conditions, *DAAM2* mRNA expression was significantly increased in both term cytotrophoblasts (p = 0.005; Fig. 4a) and term placental explants (p = 0.040; Fig. 4b), compared to control cells and tissues cultured under normoxic conditions.

### Knockdown of DAAM2 in primary cytotrophoblasts

**Figure 5:**
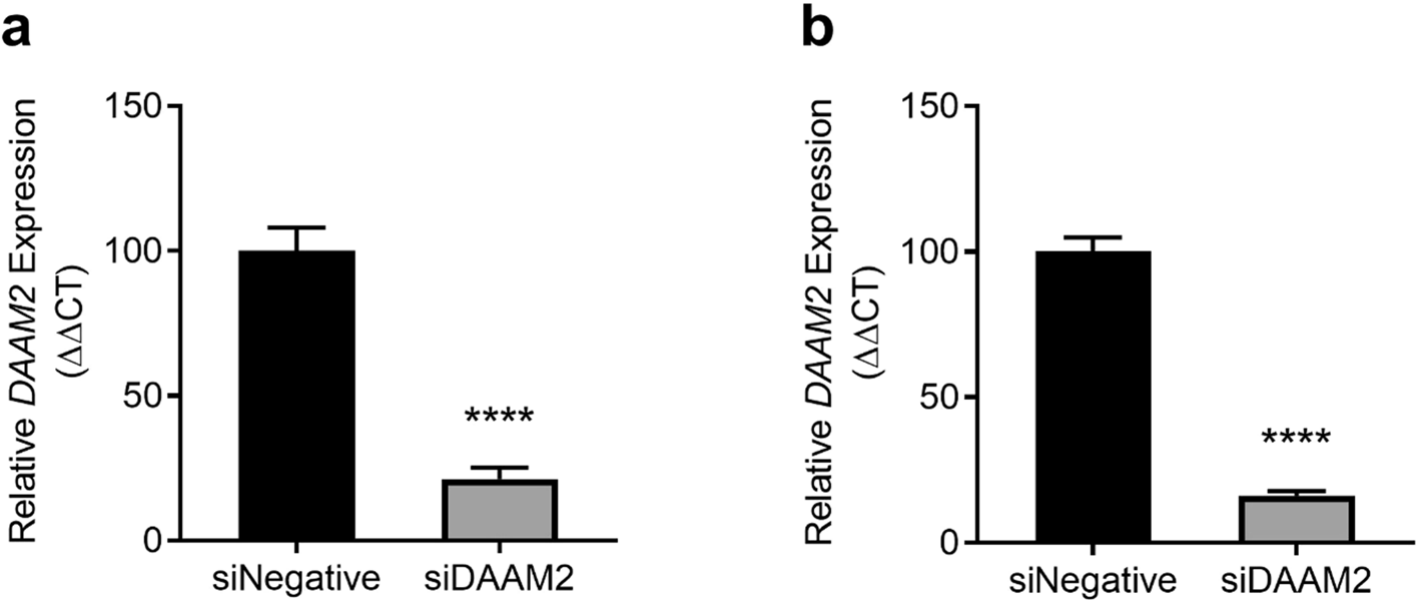
Knockdown of *DAAM2* under normoxic (8% O_2_) and hypoxic (1% O_2_) conditions. Cytotrophoblast expression of *DAAM2* mRNA was significantly knocked down under both normoxic (A) and hypoxic conditions with siRNA against *DAAM2* (siDAAM2) compared to the negative control siRNA (siNegative). Data presented as fold change from control ± SEM. ****p < 0.0001. n = 3 experimental replicates (each sample from a different patient), with each experiment performed in triplicate.

In isolated primary cytotrophoblasts, siRNA directed against *DAAM2* significantly decreased expression of *DAAM2* mRNA by approximately 80%, under both normoxic (p < 0.0001; Fig. 5a) and hypoxic (p < 0.0001; Fig. 5b) conditions. Importantly, siRNA against *DAAM2* did not negatively affect cell survival at either oxygen tension (Supplementary Fig. S4).

### Silencing DAAM2 alters expression of apoptosis and oxidative stress markers under hypoxia

To assess the potential functional roles of *DAAM2* in the placenta, we assessed expression of important genes in pathways of growth, apoptosis and oxidative stress in cytotrophoblasts where *DAAM2* had been silenced under hypoxic conditions.

**Figure 6:**
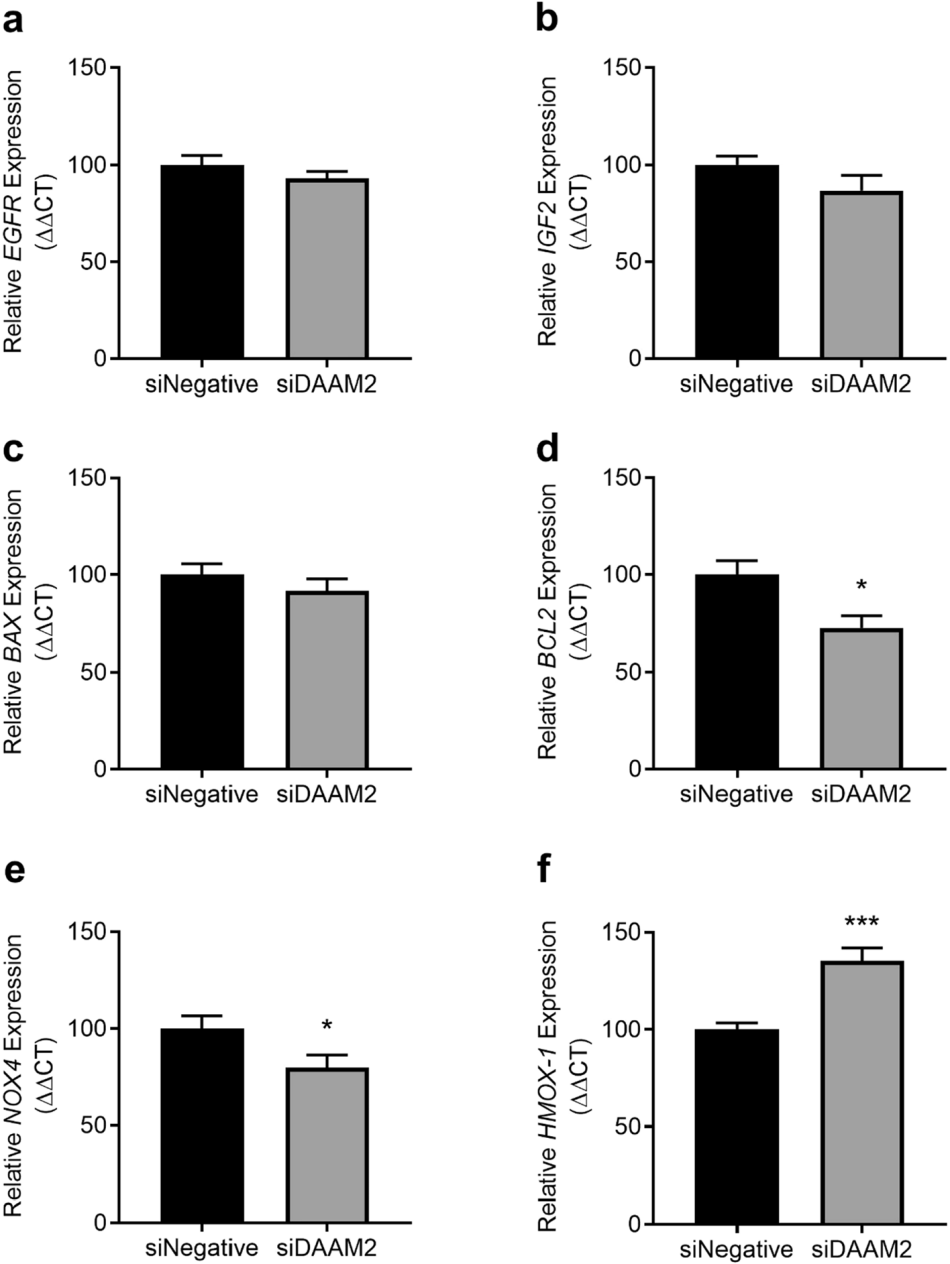
Effect of *DAAM2* knockdown on expression of cell growth, apoptosis and oxidative stress genes under hypoxia (1% O_2_). Knockdown of *DAAM2* with siRNA (siDAAM2) significantly decreased (**d**) *BCL2*, (**e**) *NOX4* and increased (**f**) *HMOX-1* mRNA expression compared to the negative siRNA control (siNegative), but had no effect on (**a**) *EGFR*, (**b**) *IGF2* or (**c**) *BAX*. Data presented as fold change from control ± SEM. *p < 0.05, ***p < 0.001. n = 3 experimental replicates (each sample from a different patient), with each experiment performed in triplicate.

Under hypoxic conditions, knockdown of *DAAM2* did not affect the expression of genes involved in placental growth and proliferation: epidermal growth factor receptor (*EGFR)* and insulin-like growth factor 2 (*IGF2)* (Fig. 6a,b, respectively). Silencing *DAAM2* did not alter expression of the pro-apoptotic gene, BCL2 Associated X (*BAX)* (Fig. 6c), but significantly decreased pro-survival B-cell lymphoma 2 (*BCL2)* mRNA expression (p = 0.011; Fig. 6d). mRNA expression of oxidative stress marker, NADPH oxidase 4 (*NOX4)* was significantly decreased with *DAAM2* knockdown compared to the negative siRNA control (p = 0.048; Fig. 6e), whilst the anti-oxidant gene, heme oxygenase 1 (*HMOX-1)* was significantly increased (p = 0.0002, Fig. 6f).

Silencing *DAAM2* did not affect expression of any of these genes under normoxic conditions (Supplementary Fig. S5).

## Discussion

In this paper, we identified that circulating *DAAM2* mRNA has potential to detect fetal growth restriction (FGR), is expressed in the human placenta throughout gestation and is dysregulated with hypoxia and in disease settings. Furthermore, silencing *DAAM2* alters stress markers in the placenta.

DAAM2 is a key regulator of the Wnt signaling pathway, an ancient and evolutionarily conserved pathway that regulates crucial aspects of cell fate determination, cell migration, cell polarity, neural patterning and organogenesis during embryonic development^13,14^. The first study to report on *Daam2* in the placenta was published recently, demonstrating a potential role in placental vascularization and the establishment of the maternal–fetal blood supply in mice^15^. Importantly, there are no published studies investigating DAAM2 function in the *human* placenta.

Previously, we identified increased *DAAM2* mRNA in the circulation of women whose pregnancies were complicated by early onset fetal growth restriction by next generation sequencing^12^. However, in that report we did not explore *DAAM2* expression further as a marker for fetal growth restriction as we focused on other genes, nor did we perform mechanistic studies in the placenta or explore the potential source for the elevated mRNA in the maternal circulation.

In this study, we performed a further analysis of circulating *DAAM2* mRNA concentrations where we characterised the diagnostic potential of *DAAM2*. We found that circulating *DAAM2* mRNA identified preterm FGR with an area under the ROC curve (AUC) of 0.83. This suggests *DAAM2* may have potential to predict the risk of preterm FGR. However, as noted in our prior report, a possible limitation as a clinical biomarker is that circulating levels of *DAAM2* mRNA may be altered by the administration of corticosteroids^12^. Thus, further studies are needed to determine whether this differential expression in the circulation remains in a population that has not received corticosteroids.

Regardless of biomarker status, our data (combined with the recent report in animal models showing the gene plays an important role in placental development^15^) suggest DAAM2 may be involved in placental development, fetal growth and the pathological condition of fetal growth restriction. The current study demonstrates for the first time that *DAAM2* is expressed by the human placenta. We confirmed expression of *DAAM2* in first trimester, second trimester and term placenta, but interestingly found that *DAAM2* expression was highest at term. *DAAM2* expression was detected as early as seven weeks gestation, suggesting a possible role for DAAM2 in early placental vascularistion and establishment of the maternal–fetal blood supply, as seen in mice^15^. Both mouse and human placenta are hemochorial, and both contain fetal capillaries surrounded by layers of trophoblasts directly bathing in maternal blood^18^. However, there are also key differences between mouse and human placental vascularisation, thus further investigation is needed to explore the potential role for DAAM2 in the first trimester.

Consistent with our discovery in the maternal circulation, we identified an increase in DAAM2 protein in human placental tissue from pregnancies complicated by early onset fetal growth restriction. However, no differences were detected in placental *DAAM2* mRNA expression. It is important to note that in the placental samples available to examine *DAAM2* mRNA expression, there was a significant difference in gestational age between our controls and fetal growth restriction-complicated pregnancies. It is difficult to ascertain whether this difference is clinically significant, but given we identified a difference in *DAAM2* expression across gestation, this may confound interpretation of the mRNA findings. Examination of a larger cohort of early onset cases and gestation-matched controls would be of value to clarify these discrepant findings. Additionally, as with all studies, mRNA expression is not always translated to protein production, hence may be a point of difference. Importantly, in these placental studies we assessed DAAM2 protein and mRNA expression in samples where corticosteroids were given in both the controls and pregnancies complicated by fetal growth restriction. Therefore, the finding of increased production of DAAM2 in the control and fetal growth restricted placentas are not confounded by corticosteroid administration. The control placentas used in these studies were carefully selected, minimising confounding effects. However, given the control placental tissue was obtained from preterm deliveries, it remains that they are not perfect controls.

Placental hypoxia plays an important role in placental dysfunction, and consequently is a contributing factor to the pathophysiology of fetal growth restriction^6^. Accordingly, we examined *DAAM2* expression in the placenta under hypoxic conditions, finding that *DAAM2* mRNA expression was increased in both isolated cytotrophoblasts and placental explant tissue. This suggests that hypoxia regulates *DAAM2* expression, and is consistent with the dramatically increased *DAAM2* mRNA in the maternal circulation of pregnancies complicated by severe fetal growth restriction^12^.

Given our finding that DAAM2 was increased in the dysfunctional placenta, we examined whether reducing its expression could be beneficial and confer protection or enhance expression of growth associated genes. Silencing *DAAM2* expression in primary cytotrophoblasts under hypoxia did not impair cell viability, suggesting that DAAM2 is not essential for trophoblast cell survival. Loss of *DAAM2* did not alter expression of *IGF2* and *EGFR*, key genes whose dysregulation has been found to be associated with impaired placental development and fetal growth restriction^19–21^, thus *DAAM2* is unlikely to be driving these pathways. Additionally, the pro-apoptotic gene *BAX*^22^ was not altered with *DAAM2* suppression. However, silencing *DAAM2* decreased expression of the pro-survival gene, *BCL2*. It is important to note that in addition to its pro-survival role, BCL2 also acts through non-canonical pathways, including regulation of mitrochondrial membrane permeabilization and oxidative stress^23,24^. It is therefore unsurprising that silencing *DAAM2* also altered expression of *NOX4*, a marker of oxidative stress^25^. Silencing *DAAM2* also increased mRNA expression of the cytoprotective antioxidant enzyme *HMOX-1*^26–28^. Decreasing oxidative stress in the placenta may have important benefits, especially when hypoxia is driving damage and dysfunction. These findings suggest DAAM2 may have an important role in placental dysfunction, and suppressing DAAM2 in the placenta could be beneficial. Future studies examining the effect of excess *DAAM2* may facilitate our understanding of the function of DAAM2 in the placenta.

While a clear role for DAAM2 in the human placenta is not yet apparent, collectively these data and the identification of *Daam2* in the murine placenta^15^ suggest important roles for DAAM2 in the placenta.

In this report, we demonstrated increases in DAAM2 expression in placentas complicated by early onset fetal growth restriction and hypoxia, indicating a potential role in the dysfunctional placenta. Additionally, we identified that placental expression of *DAAM2* increases with advancing gestation, and that suppression of *DAAM2* enhanced cytoprotective gene pathways in hypoxic cytotrophoblasts. A strength of this study is the use of prized clinical samples, and collaboration with clinical expertise. Another strength of this study is the assessment of DAAM2 in primary placental cells and tissues, rather than cell lines. However, further studies are required to expand these findings and uncover the role of DAAM2 in the healthy and dysfunctional placenta.

## Methods

### Fetal OXygenation (FOX) Study

Maternal peripheral blood was collected as part of the FOX Study as previously described^12^. In summary, blood was collected from 128 women with preterm growth restricted fetuses and from 42 women at matched gestations (28 and 34 weeks) with appropriately grown fetuses that progressed to birth at term, across six tertiary hospitals (in Australia and New Zealand). Table 1 provides the baseline clinical characteristics of study participants in the FOX study. Samples were collected directly into PAXgene Blood RNA tubes (Pre-Analytix, Hombrechtikon, Switzerland) to maintain nucleic acid stability and processed according to manufacturer’s instructions. All blood samples were collected after corticosteroid administration, immediately prior to delivery.

Ethical approval was obtained from all institutions (Approval numbers: MHW R11/04, RWH + Sunshine Hospital 10/41, MMH 1928M, RHW 12/240, RNSH 1305-151M, NWH, ACH 12/NTA/96/AM02) and all women provided written, informed consent. Experiments were performed following the relevant institutional guidelines and regulations.

Preterm fetal growth restriction was defined as a customized birthweight < 10th centile (www.gestation.net, Australian parameters) requiring iatrogenic delivery prior to 34 weeks gestation with uteroplacental insufficiency (asymmetrical growth + abnormal artery Doppler velocimetry ± oligohydramnios ± abnormal fetal vessel velocimetry). Fetal growth restriction due to infection, chromosomal or congenital abnormalities, and multiple pregnancy was excluded.

Fetal hypoxic status in the preterm growth restricted cohort was determined by collecting umbilical artery blood at birth and measuring the pH, where hypoxia was defined as pH < 7.2, and normoxia as pH ≥ 7.2.

### Placental tissue collection

Ethical approval was obtained from the Mercy Health Human Research Ethics Committee (R11/34) and Austin Health Human Research Ethics Committee (HREC/18/Austin/44). Women presenting to the Mercy Hospital for Women (Heidelberg, Victoria) and The Northern Hospital (Epping, Victoria) gave informed, written consent for the collection of tissue. Women presenting to the Broadmeadows Health Service (Broadmeadows, Victoria) gave informed, written consent for the collection of conceptus samples at surgical termination of pregnancy. Experiments were performed following institutional guidelines and regulations.

First trimester placental tissue was obtained from conceptus material collected at surgical terminations of singleton pregnancies (7–10 weeks gestation) under general anaesthesia via curettage or a combination of aspiration and curettage (according to the surgeon's preference). Placental tissue was identified and isolated from conceptus material, then washed in phosphate buffered saline (PBS). Placental tissue was transferred to RNAlater for 48 h, after which the tissue was snap frozen and stored at − 80 °C for subsequent analysis. Patient characteristics are described in Table 2.

**Table 2:** **Patient characteristics for placental tissue used to assess** ***DAAM2*** **expression across gestation.**

	First trimester (n = 6)	Second trimester (n = 4)	Term (n = 9)
**Maternal age, years** Median (range)	29 (25–39)	23.5 (19–27)^b^	33 (27–30)^b^
**Gestational age at sample collection, weeks** Median (IQR)	8.6 (7.55–8.875)^a,c^	27.25 (24.95–28.2)^a,b^	39.10 (39.0–39.3)^b,c^
**Body mass index (kg/m**^**2**^**)** Median (IQR)	23.47 (21.83–26.94)	40.90 (24–41)	26.22 (24.05–29.40)
**Parity no**			
0	2	0	1
1	3	4	4
≥ 2	1	0	4
**Mode of delivery**			
Vaginal	–	0	0
Caesarean Section	–	4	9
**Birth weight (g)** Median (IQR)	–	935 (736.3–1225)^b^	3360 (3050–3560)^b^

BMI data unavailable for n = 1 second trimester sample.

^a^Significant difference between first trimester and second trimester samples.

^b^Significant difference between second trimester and term samples.

^c^Significant difference between first trimester and term samples.

Placentas were obtained from cases of preterm fetal growth restriction (delivery ≤ 34 weeks gestation), defined as customized birth weight < 10th centile according to Australian population charts^29^. Cases associated with congenital infection, chromosomal or congenital abnormalities, multiple pregnancies and preeclampsia were excluded.

Control healthy, term (delivery 37–40 weeks gestation) and preterm placentas (delivery ≤ 34 weeks gestation) were collected from normotensive pregnancies where a fetus of normal customized birth weight centile (> 10th centile relative to gestation) was delivered. Placentas with evidence of chorioamnionitis (confirmed by placental histopathology) were excluded.

Term and preterm placental tissue was collected within 30 min of delivery. Preterm delivery in our controls was predominantly for iatrogenic conditions including vasa previa, suspected placental abruption and fetal anaemia. For preterm (fetal growth restriction and control) tissue collection, samples from four sites of the placenta were washed in ice cold PBS and preserved in RNAlater for 48 h, after which the tissue was snap frozen and stored at − 80 °C for subsequent analysis. Patient characteristics are described in Tables 2, 3 and 4.

**Table 3:** **Patient characteristics of women with fetal growth restriction and control samples for gene (mRNA) expression studies.**

	Preterm controls (n = 10)	Fetal growth restriction (n = 14)
**Maternal age, years** Median (IQR)	34 (26.5–37.5)	30 (25.3–33.5)
**Gestational age at delivery, weeks** Median (IQR)	30 (29.4–31.6)	32.7 (30.9–34.0)*
**Body mass index (kg/m**^**2**^**)** Median (IQR)	28.4 (24.0–30.0)	25.8 (18.75–29.5)
**Parity no. (%)**		
0	2 (20.0)	9 (64.3)
1	4 (40.0)	2 (14.3)
≥ 2	4 (40.0)	3 (21.4)
**Highest SBP prior to delivery (mmHg)** Median (IQR)	120 (110–126.3)	120 (115–126.3)
**Highest DBP prior to delivery (mmHg)** Median (IQR)	70 (67.5–76.25)	76.5 (70–83.5)*
**Mode of delivery**		
Vaginal (%)	0 (0)	0 (0)
Caesarean Section (%)	10 (100)	14 (100)
**Birth weight (g)** Median (IQR)	1496 (1322–2011)	1182 (973–1658)

BMI data unavailable for n = 3 preterm controls, n = 1 fetal growth restriction sample.

*p < 0.05.

**Table 4:** **Patient characteristics of women with fetal growth restriction and gestation matched control samples for protein studies.**

	Preterm controls (n = 16)	Fetal growth restriction (n = 9)
**Maternal age, years** Median (IQR)	28.5 (25.25–36.75)	29 (21–32.5)
**Gestational age at delivery, weeks** Median (IQR)	30 (29.4–32.08)	31.4 (30.7–33.3)
**Body mass index (kg/m**^**2**^**)** Median (IQR)	26 (21.65–32.15)	20 (18.25–27.7)
**Parity no. (%)**		
0	5 (33.3)	7 (77.8)
1	8 (53.3)	1 (11.1)
≥ 2	2 (13.3)	1 (11.1)
**Highest SBP prior to delivery (mmHg)** Median (IQR)	120 (119.3–130.0)	120 (113.5–127.5)
**Highest DBP prior to delivery (mmHg)** Median (IQR)	70 (63.5–78.75)	78 (70–81.5)
**Mode of delivery**		
Vaginal (%)	3 (20)	0 (0)
Caesarean Section (%)	12 (80)	9 (100)
**Birth weight (g)** Median (IQR)	1587 (1277–1976)	1000 (893.0–1375)*

BMI data unavailable for n = 3 preterm control samples.

*p < 0.05.

### Placental explant isolation and culture

Placentas were obtained from normal term pregnancies (> 37 weeks gestation) at elective Caesarean section for explant dissection. Placental explants were isolated with maternal and fetal surfaces removed. Three small pieces of placenta totalling 10–15 mg of tissue per well were cultured in 24-well plates, each containing media made up of Gibco Dulbecco's Modified Eagle Medium (DMEM; ThermoFisher Scientific, Scoresby, Vic), supplemented with 10% fetal calf serum (FCS; Sigma-Aldrich, St Louis, USA) and 1% Anti-Anti (AA; Life Technologies, Carlsbad, CA, USA). Explants were cultured under 8% O_2_, 5% CO_2_ at 37 °C overnight (16–18 h). After replacement with fresh media (DMEM, 10% FCS, 1%AA), explant tissue was cultured at 37 °C for 24 h under 8% O_2_ (normoxic conditions) or 1% O_2_ (hypoxia). Following this, explant tissue was weighed, snap frozen and stored at − 80 °C for subsequent analysis.

### Primary cytotrophoblast isolation and culture

Human primary cytotrophoblasts were isolated from normal term placentas from elective Caesarean section as previously described^30^. The cells were plated in media (DMEM, 10% FCS, 1%AA) on fibronectin (10 µg/mL; BD Bioscience, USA) coated culture plates. Viable cells were incubated under 8% O_2_, 5% CO_2_ at 37 °C overnight to equilibrate. After replacement with fresh media, cytotrophoblasts were cultured at 37 °C for 24 h under 8% O_2_ (normoxic conditions) or 1% O_2_ (hypoxia). Following this, cells were collected for RNA extraction.

### Silencing genes in primary cytotrophoblasts

Small interfering RNA (siRNA) designed against *DAAM2* (M-014010-00-0005; Dharmacon, Lafayette, CA, USA) or a negative siRNA control (Qiagen, Valencia, CA, USA) were combined with lipofectamine (RNAiMax; Invitrogen) in optimem (ThermoFisher Scientific) to complex for 20 min at room temperature. After equilibration of the isolated cytotrophoblasts overnight (described above), fresh trophoblast media (DMEM, 10% FCS, no AA) was added to each well and siRNA complexes added in a dropwise manner. Cytotrophoblast cells with siRNA were cultured at 37 °C for 48 h under 8% O_2_ (normoxic conditions) or 1% O_2_ (hypoxia). Following this, media and cells were collected for subsequent analysis.

### MTS cell viability assay

Cell viability was assessed following siRNA treatment using the MTS assay, CellTiter 96-AQueous One Solution (Promega, Madison WI) according to manufacturer instructions.

### Quantitative real time polymerase chain reaction (qPCR)

Total RNA was extracted from whole blood via PAXgene Blood miRNA Kit (Pre-Analytix, Hombrechtikon, Switzerland) according to manufacturer’s instructions, as described previously^12^. RNA was extracted from placental tissue (collected from first trimester, preterm and term gestations), cultured explants and isolated primary cytotrophoblasts using the Qiagen RNeasy Mini Kit, according to the manufacturer’s instructions. RNA concentration was quantified using a Nanodrop 2000 spectrophotometer (ThermoFisher Scientific, Waltham, MA). RNA was converted to cDNA using the Applied Biosystems High-Capacity cDNA Reverse Transcription Kit (Thermofisher), as per manufacturer’s instructions on the iCycler iQ5 (Biorad) or MiniAmp Thermal Cycler (Applied Biosystems, CA, USA). Quantitative Taqman PCR was performed to quantify mRNA expression of *DAAM2, BAX, BCL2, EGFR, IGF2, NOX4* and *HMOX-1* (Hs00322497_m1, Hs00180269_m1, Hs00608023_m1, Hs01076078_m1, Hs04188276_m1, Hs00418356_m1, and Hs01110250_m1 respectively; Life Technologies), as well as reference genes for blood: *YHWAZ*, *B2M* and *GUSB* (Hs01122454_m1, Hs00187842_m1, Hs00939627_m1; Life Technologies), cytotrophoblast cells: *YWHAZ* and explants and placental tissue: *TOP1* and *CYC1* (Hs01122454_m1, Hs00243257_m1, and Hs00357717_m1 respectively; Life Technologies). Stability of reference genes was confirmed for each tissue type and used appropriately. Taqman qPCR was performed on the CFX384 (Biorad) or QuantStudio 5 (Applied Biosystems) with the following run conditions: 50 °C for 2 min, 95 °C for 10 min, 95 °C for 15 s, 60 °C for 1 min or 50 °C for 2 min, 95 °C for 20 s, 95 °C for 3 s, 60 °C for 30 s (40 cycles). All data were normalized to the appropriate reference gene as an internal control and calibrated against the average Ct of the control samples. All cDNA samples were run in duplicate.

### Western blot analysis

Protein lysates were extracted from placental tissue from early onset preterm fetal growth restricted pregnancies (≤ 34 weeks) using RIPA lysis buffer containing proteinase and phosphatase inhibitors (Sigma Aldrich). Protein concentrations were assessed with Pierce BCA Protein Assay Kit (ThermoFisher Scientific). Placental lysates (20 µg) were separated on 10% gels and PVDF membranes (Millipore; Billerica, MA, United States). Membranes were blocked with 5% skim milk, prior to overnight incubation with DAAM2 primary antibody at 1:500 in 5% skim milk/TBS-T (GTX33141, Sapphire Bioscience, NSW, Australia). Blots were incubated with anti-rabbit secondary antibody at 1:2500 in 5% skim milk for 1 h (W401; Promega, VIC, Australia). Membranes were developed with enhanced chemiluminescence reagent (GE Healthcare Life Sciences, NSW, Australia) and detected using the ChemiDoc XRS (BioRad). β-actin acted as the loading control at 1:20,000 in 5% skim milk (Santa Cruz, Texas, USA). Densitometry was performed on images of the blots using ImageJ software (NIH, Bethesda, MD, USA).

### Statistical analysis

All in vitro experiments were performed with technical triplicates and repeated with n ≥ 3 different patient samples. Data were tested for normal distribution and statistically tested as appropriate. Either an unpaired t-test (parametric) or Mann–Whitney test (non-parametric) was used. The area under the receiver operating curve (AUC) was calculated to determine the sensitivity/specificity performance for *DAAM2*. All data are expressed as mean ± SEM. P-values < 0.05 were considered significant. Statistical analysis was performed using GraphPad Prism 8 software (GraphPad Software, Inc.; San Diego, CA, USA).

## Supplementary Information

Supplementary Information.

## Data Availability

The datasets generated during and analysed during the current study are available from the corresponding author on reasonable request.
