# Supplementary material for: DAAM2 is elevated in the circulation and placenta in pregnancies complicated by fetal growth restriction and is regulated by hypoxia
Source: Sci Rep. 2021 Mar 10;11:5540. doi: 10.1038/s41598-021-84785-7 (PMC7946951; doi:10.1038/s41598-021-84785-7)
Supplement: Supplementary file 1 — Supplementary Information. [file 41598_2021_84785_MOESM1_ESM.docx]

**Supplementary File**

**DAAM2 is elevated in the circulation and placenta in pregnancies complicated by fetal growth restriction and is regulated by hypoxia.**

**Authors**: Natasha de Alwis, Sally Beard, Natalie K. Binder, Natasha Pritchard, Tu’uhevaha J. Kaitu’u-Lino, Susan P. Walker, Owen Stock, Katie Groom, Scott Petersen, Amanda Henry, Joanne M. Said, Sean Seeho, Stefan C. Kane, Lisa Hui, Stephen Tong, and Natalie J. Hannan*


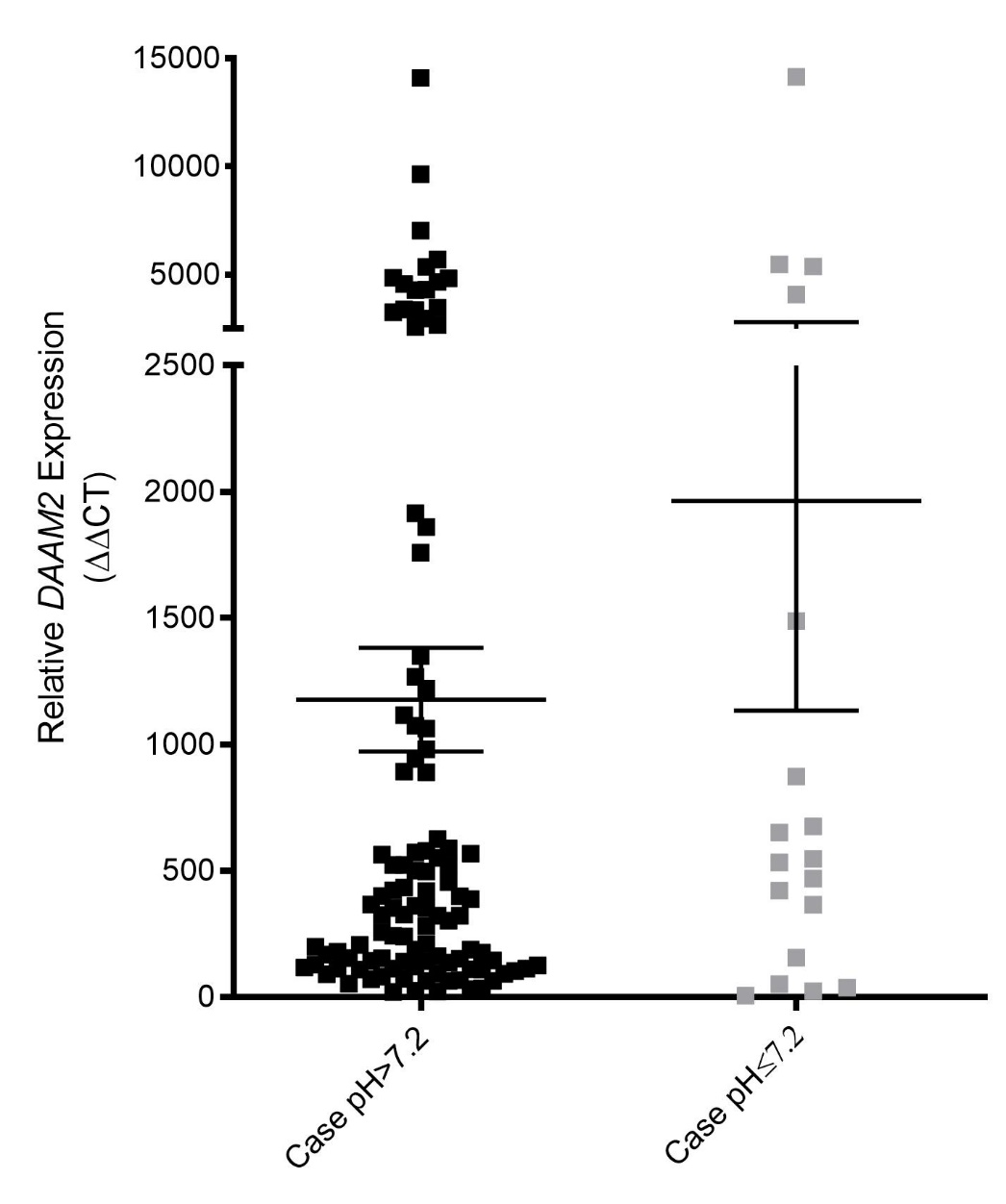


**Supplementary Figure S1. *DAAM2* mRNA in the maternal circulation from pregnancies complicated by preterm fetal growth restriction (FGR), separated by umbilical artery blood pH.** *DAAM2* mRNA is not altered in cases with umbilical artery blood pH≤7.2 compared to cases with pH>7.2. Data presented as fold change from control ± SEM.

**
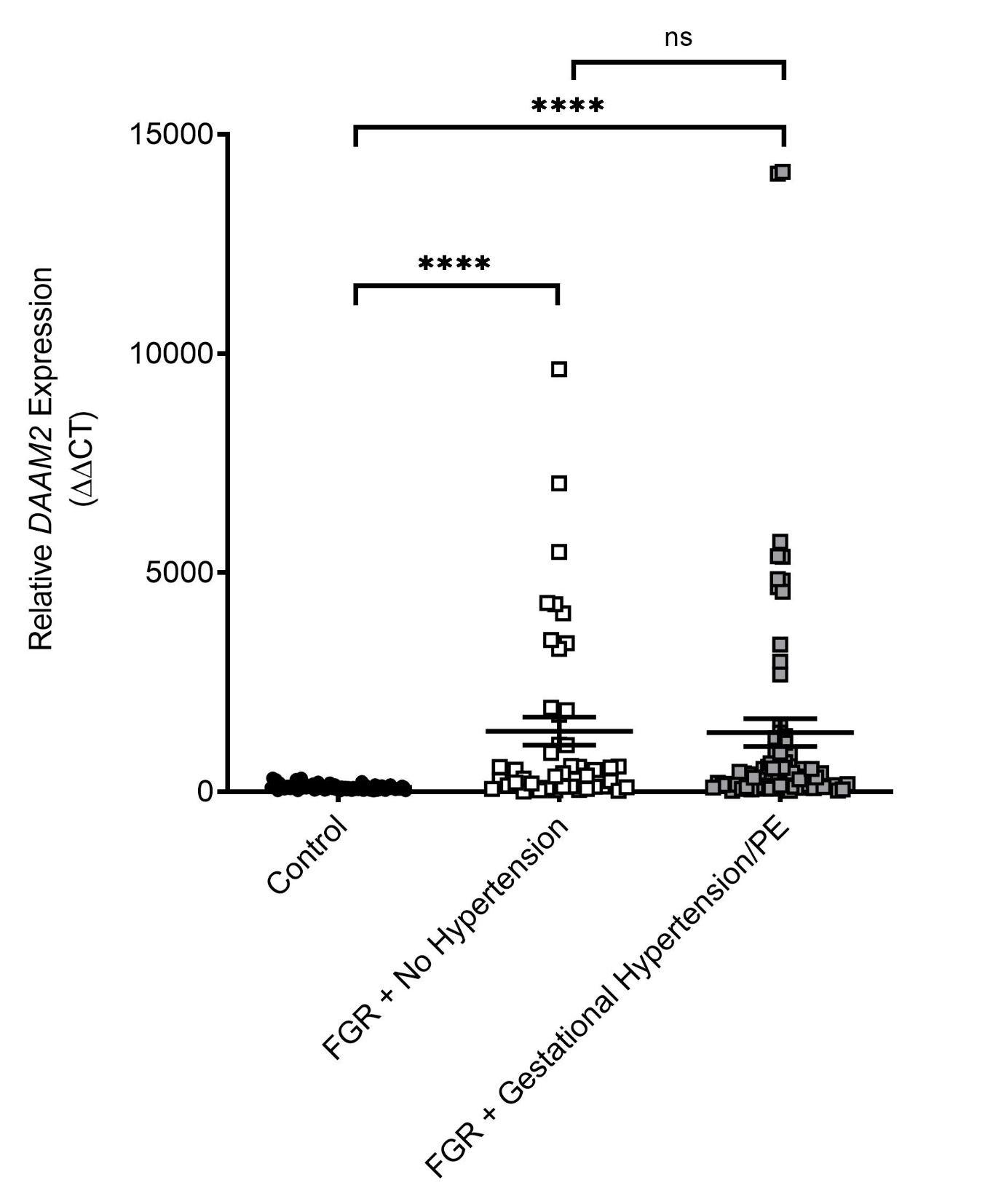
Supplementary Figure S2. *DAAM2* mRNA in the maternal circulation from pregnancies complicated by preterm fetal growth restriction (FGR), separated by presence of gestational hypertension or preeclampsia (PE) (FOX Study).** *DAAM2* mRNA is significantly different in cases compared to control, irrespective of coexistent hypertension. There is no significant difference in *DAAM2* expression in the cases with or without hypertension or PE. Data presented as fold change from control ± SEM. ****p<0·0001.


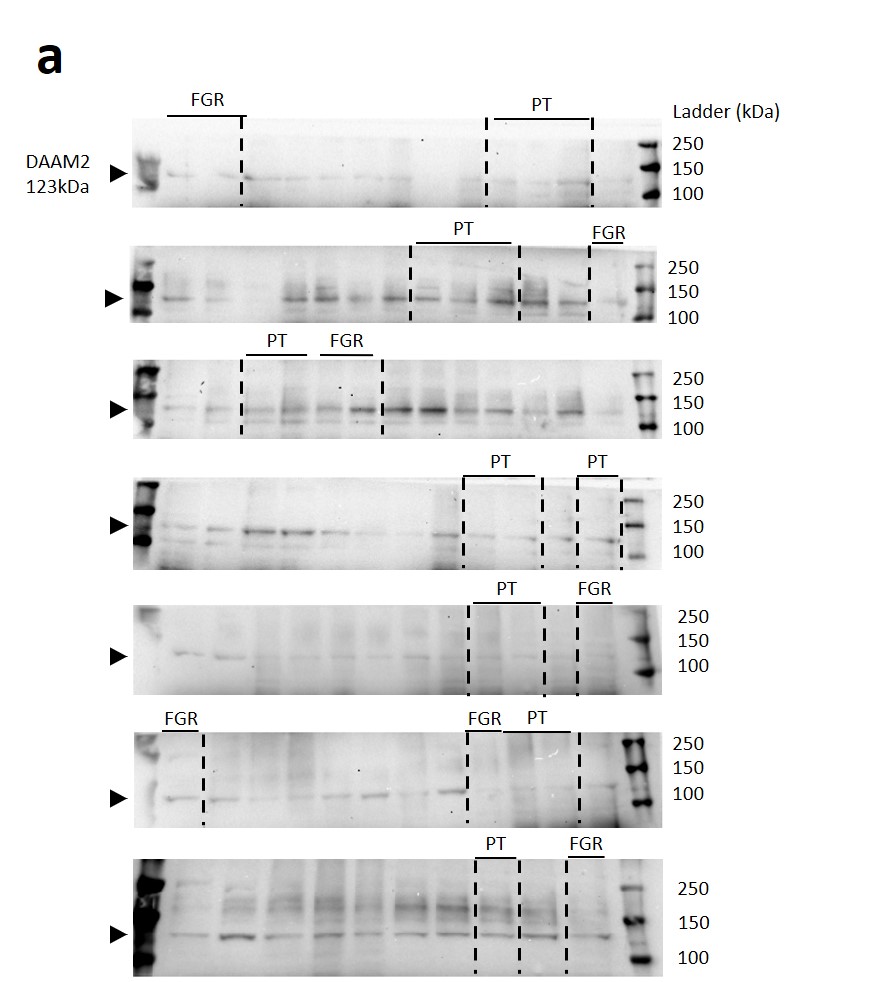

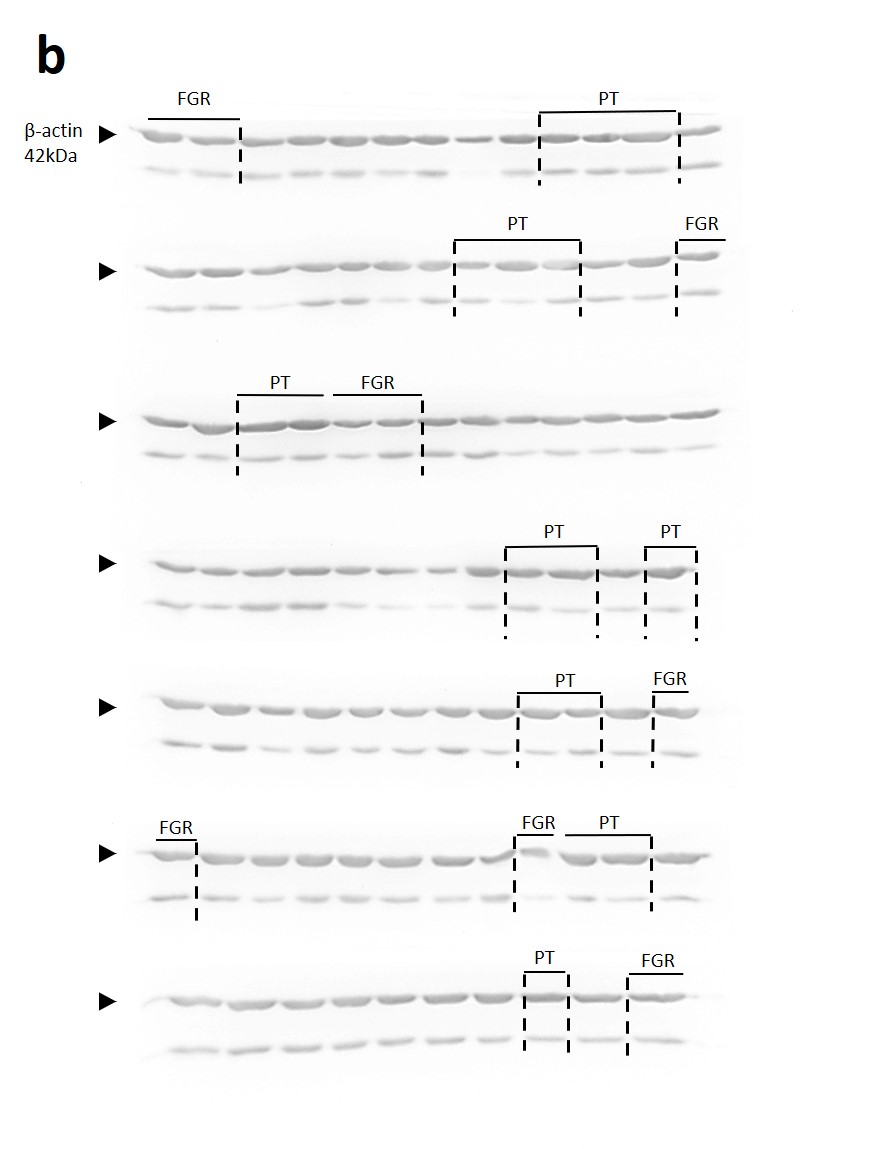


**Supplementary Figure S3. Western blot images of DAAM2 protein in fetal growth restricted (n=9) and preterm (n=16) control placental tissue (≤34 weeks).** a) DAAM2 protein b) β-actin protein (reference). Bands of interest are marked (►). These blots were used to derive the densitometric data shown in Figure 3c and used to create a representative cropped image for Figure 3b. Lanes where patients are not relevant to this study are demarcated with dashed lines and were excluded from densitometric analysis. The samples labelled were included in analysis; PT – preterm, FGR – fetal growth restriction.

**
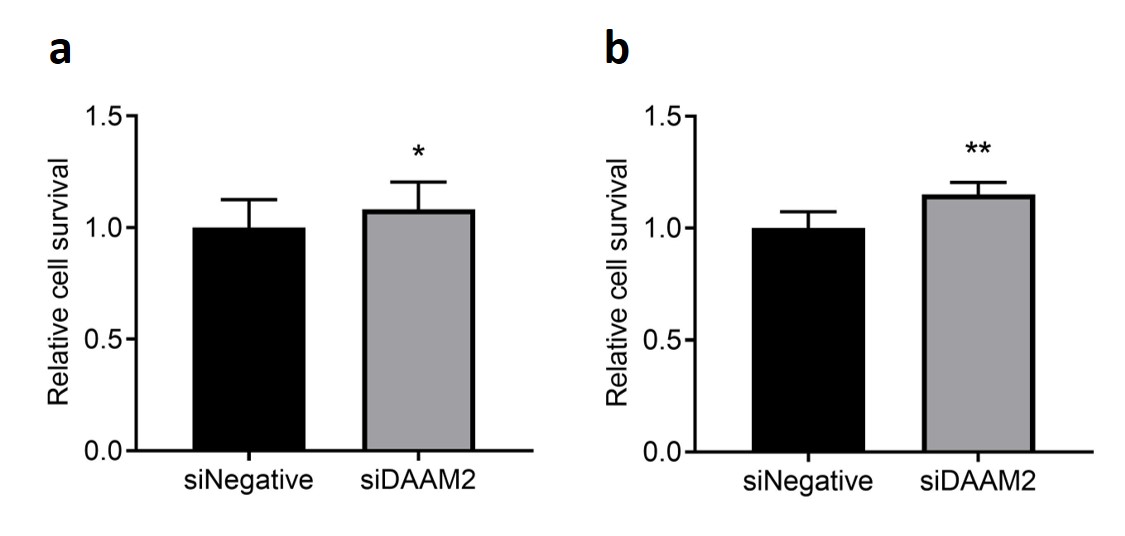
**

**Supplementary Figure S4. Cell survival with addition of silencing siRNAs under a) normoxic (8% O_2_) and hypoxic (1% O_2_) conditions.** MTS assay demonstrated significantly increased cell survival with knockdown of *DAAM2* under both oxygen tensions. Data presented as fold change from control ± SEM. *p<0·05, **p<0·01. n=3 experimental replicates, with each sample from a different patient. Each experiment was run in triplicate.


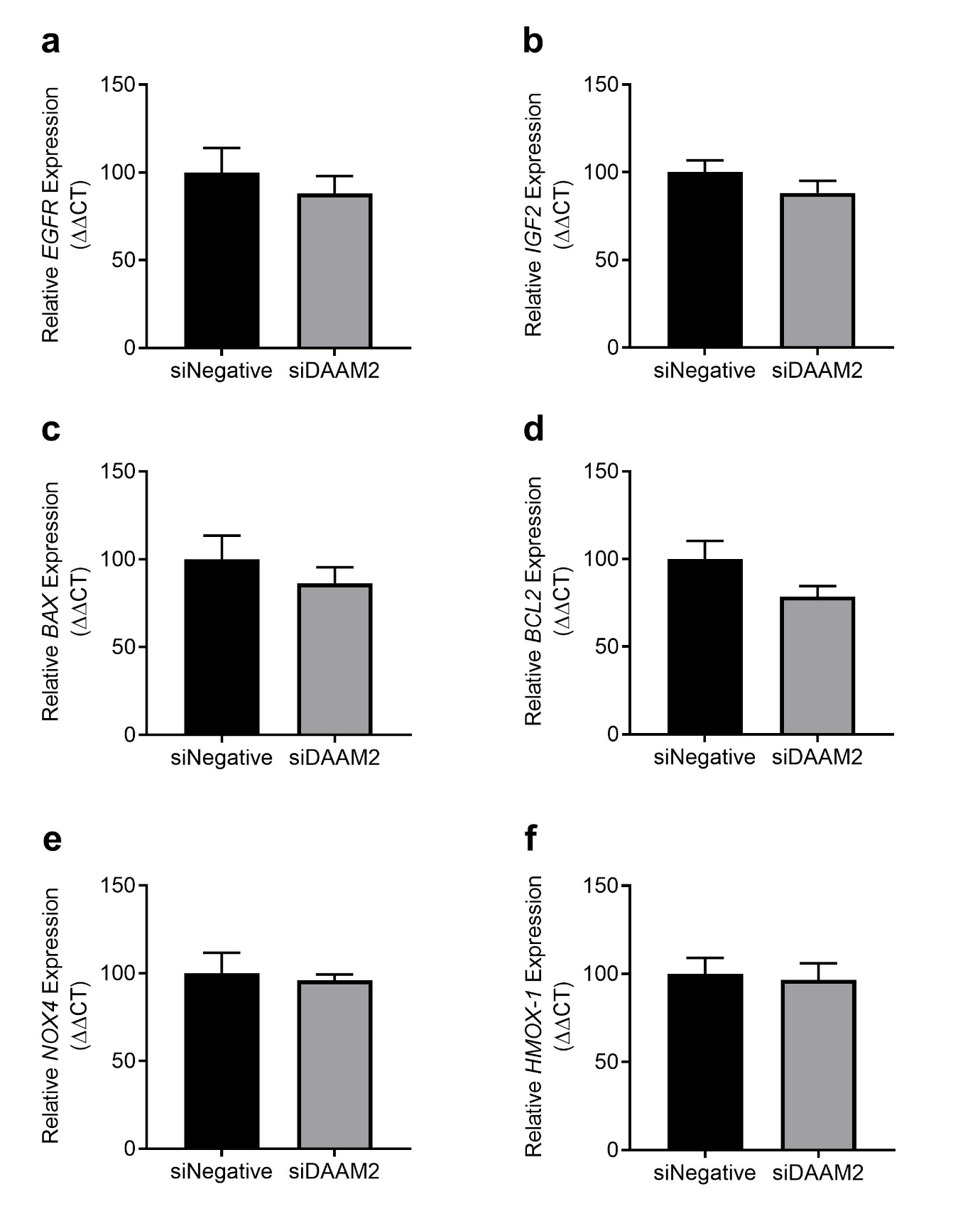


**Supplementary Figure S5. Effect of silencing *DAAM2* on expression of cell growth, apoptosis and oxidative stress genes under normoxic conditions (8% O_2_) assessed by qPCR.** Expression of all genes was unchanged with *DAAM2* knockdown compared to negative control. Data presented as fold change from control ± SEM. n=3 experimental replicates, with each sample from a different patient. Each experiment was run in triplicate.
